# Plant Cell Wall Proteins and Development

**DOI:** 10.3390/ijms21082731

**Published:** 2020-04-15

**Authors:** Elisabeth Jamet, Christophe Dunand

**Affiliations:** Laboratoire de Recherche en Sciences Végétales, Université de Toulouse, CNRS, UPS, 31320 Auzeville Tolosane, France

Plant cell walls surround cells and provide both external protection and a means of cell-to-cell communication. They mainly comprise polymers like polysaccharides (cellulose, hemicelluloses, and pectins) and lignin in lignified secondary walls and a small amount of cell wall proteins (CWPs) [1,2]. CWPs are major players of cell wall remodeling and signaling. Cell wall proteomics, as well as numerous genetic or biochemical studies, have revealed the high diversity of CWPs, among which proteins acting on polysaccharides, proteases, oxido-reductases, lipid-related proteins, and structural proteins ([3,4,5,6,7]). CWPs may have enzymatic activities such as cutting/ligating polymers or processing/degrading proteins [8]. They may also contribute to the supra-molecular assembly of cell walls via protein/protein or protein/polysaccharide interactions [9,10,11]. Thanks to these biochemical activities, they contribute to the dynamics and functionality of cell walls. Even though much research has already been pursued to shed light on the many roles of CWPs, many functions still remain to be discovered, especially for proteins identified in cell wall proteomes with yet unknown function.

This Special Issue “Plant Cell Wall Proteins and Development” has welcomed a selection of articles in the field of cell wall biology, which were focused on cell wall proteins and their roles during development. Eight experimental articles, nine up-to-date review articles, as well as a concept article, have been published. We wish to thank all the authors for their great contribution to this unique collection of articles as well as the International Journal of Molecular Science supporting team. 

The content of this Special Issue embraces several topics, all of them stressing the roles of cell wall proteins: cell wall proteomics studies on monocot species [7,12]; the role of cell wall proteins during plant development [13,14,15] or in response to environmental stresses [16,17,18,19]; overviews on several cell wall protein families either from green microalgae [20] or from plants, i.e., fasciclin arabinogalactan proteins (FLAs) [21,22], membrane-bound class III peroxidases (Class III Prxs) [23], pectin methylesterases inhibitors [24], DUF642 (Domain of Unknown Function 642) proteins [25], and Proline-rich, Arabinogalactan proteins, conserved Cysteines (PAC) domain-proteins [26]; and the role of fasciclin arabinogalactan proteins (FLAs) in Ca^2+^ signaling during plant morphogenesis [27,28].

For two decades, cell wall proteomics has become a powerful experimental approach and has revealed the diversity of the cell wall protein families. *Arabidopsis thaliana* has been the most studied plant species, and almost half of its expected cell wall proteome has been described so far (see *WallProtDB*, http://www.polebio.lrsv.ups-tlse.fr/WallProtDB/). The monocotyledon species have been studied more recently thanks to the sequencing of additional genomes like those of *Oryza sativa* [29], *Brachypodium distachyon* [30], and *Triticum aestivum* [31] as well as the availability of transcriptomics data as for *Saccharum* spp [32]. Calderan-Rodrigues et al. [7] provide a comparison of monocotyledon and dicotyledon cell wall proteomes and have discussed the specificities of the former. Such specificities were related to the differences between the composition and structure of monocotyledon and dicotyledon cell walls [1,33]. Also, Cherkaoui et al. [12] report on the comparison between cell wall proteomes of the endosperm, and the outer layers of the wheat grain. They reveal a strong metabolic activity in the cell wall during endosperm differentiation, whereas the accumulation of proteins was more important at an earlier stage of development in the outer layers.

As mentioned above, the cell wall composition and structure varys during development, and these changes can allow further differentiation processes. Betekhtin et al. [13] provide a fine mapping of cell wall epitopes in zygotic embryos of *B. distachyon* at a mature stage of development, including antibodies recognizing extensins and arabinogalactan proteins (AGPs), which are structural proteins involved in the cell wall architecture and proteins assumed to be involved in signaling, respectively. The plasma membrane is the interface between the cytoplasm and the cell wall. Its composition can vary locally in the domains characterized by particular lipid compositions. Kubátová et al. [15] show that two plasma membrane domains with a distinct lipid composition are located close to the Ortmannian ring, a cell wall domain-specific to trichomes. These plasma membrane domains are generated thanks to exocysts complex containing EXO70 subunits recognizing the target membrane. Cell-to-cell communication can be ensured through plasmodesmata [34]. Han et al. [14] provide a review article focusing on the cytoskeleton and on plasmodesmata-associated cell wall proteins like callose synthase and callose hydrolase, which are involved in the regulation of plasmodesmata closure.

Environmental cues induce modifications of the cell wall. In particular, nutrient availability can regulate cell wall composition. The absorption of nutrients by roots occurs through the apoplastic pathway. This pathway is blocked by the deposition of lignin and later of suberin at the level of the Casparian strips around endodermis cells in differentiated roots. In their review article, Ogden et al. [19] focus on the changes observed in the modulation of the suberization of the root endodermal walls in response to nutrient availability, showing that the plasticity of suberin accumulation is an adaptative response. They also show that the availability of nitrate or phosphorus modulates the development of lateral roots and/or of root hairs and has a direct effect on the transcription of genes encoding proteins involved in the biosynthesis of cell wall components or regulating the oxidative status in the cell wall. Wu et al. [18] focus on a few cell wall proteins playing critical roles during phosphorus deficiency such as expansins, Pro-rich proteins, oxidoreductases, and purple acid phosphatases. Abiotic stresses like flooding or temperature can also induce changes in the cell wall. Song et al. [17] show that xyloglucan endotransglycosylases/hydrolases (XTHs), which are hemicelluloses remodeling enzymes *in muro*, play roles in the regulation of stress responses to flooding. Indeed, the overexpression of the *A. thaliana AtXTH31* gene in soybean plants leads to increasing of resistance to flooding. Pinski et al. [16] observe changes in the accumulation of extensin and AGP epitopes in *B. distachyon* leaves exposed to cold and hot temperature stresses.

Cell wall proteins are mostly encoded by multigene families, which can comprise a large number of members like class III Prxs [35] or pectin methyl esterase inhibitors [36] (73 and 71 members in *A. thaliana*, respectively). Each member has its own regulatory pathway during development or upon stress, and even if the proteins of a give family share the same functional domains, subtle differences can confer different biological activities. As an example, AtPrx36 plays a particular role in mucilage release because of the timely regulation of expression of its gene during seed development, and of its anchoring in a cell wall microdomain [37]. Most cell wall protein families are conserved in the green lineage. This is illustrated in four articles of this Special Issue. Guerriero et al. [20] describe a family of green microalgal cellulases. Seifert et al. [21] show the conservation of the fasciclin 1 domain (FAS1) in all the kingdoms of life, suggesting a role in the mechanisms mediating interactions between the cells and their environment. He et al. [14] describe the evolution of FLAs which are possibly involved in signaling. Nguyen-Kim et al. [26] explore the PAC domain-proteins family possibly forming non-covalent networks with polysaccharides and *O*-glycoproteins.

Since cell wall proteins families contain many members, it is interesting to consider each of them independently to fully uncover their roles in cell wall biology. Three review articles present such overviews. Lüthje and Martinez-Cortes [23] describe the sub-family of membrane-bound class III Prxs which are located at the plasma membrane or in the tonoplast and are assumed to play roles in membrane protection or repair. Wormit and Usadel [24] give an overview of the roles of pectin methylesterase inhibitors (PMEIs). These proteins participate in the regulation of the degree of methylesterification of the pectic homogalacturonans, which in turn contributes to cell adhesion, cell wall porosity, and plasticity. Finally, Cruz-Valderrama et al. [25] propose a role for the DUF642 protein family in development and in response to environmental stresses by modulating directly, or indirectly, the degree of methylation of homogalacturonans. These proteins were first described as abundant proteins in cell wall proteomes [38] and were until recently considered proteins with unknown function.

This Special issue was also open to new concepts. Two articles by Lamport et al. [27,28] propose new roles for the arabinogalactan protein (AGP) family in root and shoot morphogenesis, as well as in phyllotaxis patterning. Such molecules are actually proteoglycans with a proportion of glycans of up to 90% [39], which are assumed to play roles in signaling. However, the molecular mechanisms underlying this function were not deciphered until recently when its role as an extracellular calcium capacitor was proposed [40].

Altogether, we believe that this Special Issue will provide a collection of articles allowing both experts and newcomers in the field to get a valuable update on plant cell wall biology. A combination of research articles, reviews, and concept articles allows a survey of several topics of interest today regarding the many roles of cell wall proteins.
